# Pharmacokinetics of sulbactam-durlobactam in patients with *Acinetobacter baumannii* ventriculitis: a report of two cases

**DOI:** 10.1128/aac.00674-25

**Published:** 2025-09-22

**Authors:** Nazanin Pouya, Natalie A. Finch, Alejandro Granillo, Adarsh Bhimraj, Vincent H. Tam, William R. Miller

**Affiliations:** 1 Department of Pharmacy Practice and Translational Research, University of Houston College of Pharmacy 15507, Houston, Texas, USA; 2 Department of Pharmacy, Houston Methodist Hospital 23534 , Houston, Texas, USA; 3 Division of Infectious Diseases, Department of Internal Medicine, Houston Methodist Hospital 23534 , Houston, Texas, USA; University of California San Francisco, San Francisco, California, USA

**Keywords:** beta-lactam, beta-lactamase inhibitor, CNS penetration, gram-negative bacteria

## Abstract

Two adult patients with ventriculitis due to *Acinetobacter baumannii* were treated with intravenous sulbactam-durlobactam as a part of a combination regimen. Penetration into the central nervous system was estimated (by cerebrospinal fluid [CSF]/serum area under the curve [AUC] ratios at steady state) for sulbactam (0.47–0.64) and durlobactam (0.27–0.3), respectively. In view of mostly constant CSF concentrations, single CSF levels at steady state may offer a more convenient and clinically applicable approach for monitoring therapy.

## INTRODUCTION

Infections caused by *Acinetobacter baumannii* are a growing global concern, given the treatment challenges associated with gram-negative pathogens. Ventriculo-meningitis (VM) is a severe and potentially life-threatening complication that can develop after neurosurgery or the use of devices like external drains or shunts (1). Effective treatment requires identifying the pathogen and its resistance profile, as well as ensuring adequate antibiotic penetration into the central nervous system (CNS) for successful infection clearance. The objective of this study was to evaluate concentrations of sulbactam-durlobactam in both serum and cerebrospinal fluid (CSF) during the treatment of ventriculitis.

### First case

A 58-year-old patient was admitted for altered mental status, seizure, nausea, and vomiting less than 2 weeks after a right craniotomy for resection of recurrent oligodendroglioma. Magnetic resonance imaging on admission revealed a 0.6 cm subgaleal pseudomeningocele at the vertex of the overlying right frontal craniotomy, which was also palpable on physical exam. On admission, blood cultures were negative, and white blood cells (WBC) were elevated at 30.96 k/μL on IV dexamethasone. On hospital day 2, the patient developed a probable CSF leak near the cranial incision and hydrocephalus requiring an extraventricular drain (EVD) placement, with subsequent fevers on day 6. CSF samples obtained from the EVD on days 2 and 6 grew *A. baumannii/calcoaceticus* on culture (susceptibilities shown in Appendix 1). Creatinine clearance of the patient was estimated to be 114 mL/min by the Cockcroft-Gault method (using adjusted body weight). The patient received intravenous sulbactam-durlobactam with a dose of 2 g every 6 h and meropenem with a dose of 2 g every 8 h. Each dose was given as a prolonged infusion over 3 h from hospital days 3–34. Serial serum and CSF samples were obtained over a dosing interval at steady state on day 18 of therapy. All samples were processed within 15 min of collection and stored at −80°C until drug assays.

Repeat cultures remained negative, and CSF studies improved over the remainder of the course. The EVD was removed on hospital day 32, and 4 weeks of sulbactam-durlobactam plus meropenem was completed on day 34. The patient did well until hospital day 37 when they developed new lethargy, aphasia, and focal seizures. Lumbar puncture on hospital day 47 showed worsening pleocytosis, but CSF and blood cultures were negative. The patient was re-started on meropenem, and sulbactam-durlobactam was continued with the addition of vancomycin. Despite the availability of narrower spectrum alternatives, this was to ensure potent *A. baumannii* activity and mitigate the risk of resistance development, given the severity and critical location of the infection. Repeat imaging and CSF fluid studies on hospital day 49 demonstrated improving ventriculitis. Antimicrobial therapy was narrowed to ampicillin-sulbactam and meropenem upon transfer to a long-term care facility on hospital day 63. Unfortunately, the patient had no significant neurological recovery and transitioned to hospice on hospital day 78.

### Second case

Details of this case were reported previously (2). The creatinine clearance of the patient was estimated to be 191 mL/min by the Cockcroft-Gault method (using adjusted body weight). The patient also received intravenous sulbactam-durlobactam with a dose of 2 g every 6 h and cefiderocol with a dose of 2 g every 6 h. Each dose was given as a prolonged infusion over 3 h. Serial serum and CSF samples were obtained over a dosing interval at steady state on day 13 of therapy. This patient experienced clinical success as previously reported.

Sulbactam and durlobactam concentrations in serum, as well as CSF, were quantified using the ultra-performance liquid chromatography-tandem mass spectrometry (UPLC-MS/MS) method. Proteins were precipitated using acetonitrile with 0.1% trifluoroacetic acid. Chromatographic separation with ceftazidime (as internal standard) was completed in 5 min. Multiple reaction monitoring (MRM) in negative-ion mode identified sulbactam (m/z 232.0→140.1), durlobactam (m/z 276.1→96.9), and ceftazidime (m/z 547→468.1). Calibration curves in serum (0.5–128 mg/L) and CSF (1–64 mg/L) were linear for both agents using a linear fit after 1/*x* weighting (*r*² > 0.99).

Serum concentrations of sulbactam and durlobactam over time were characterized using ADAPT5 (University of Southern California). For each component, a one-compartment pharmacokinetic model with zero-order input was used. Using the best-fit parameter estimates, area under the curve at steady state from hour 0 to 6 (AUC_0-6_ss) was derived by dose/clearance for systemic exposure and using the median CSF drug concentration over 6 h for CNS exposure. A protein binding value of 38% and 10% was assumed for sulbactam and durlobactam, respectively, to derive free systemic drug exposures (3).

**Fig 1:**
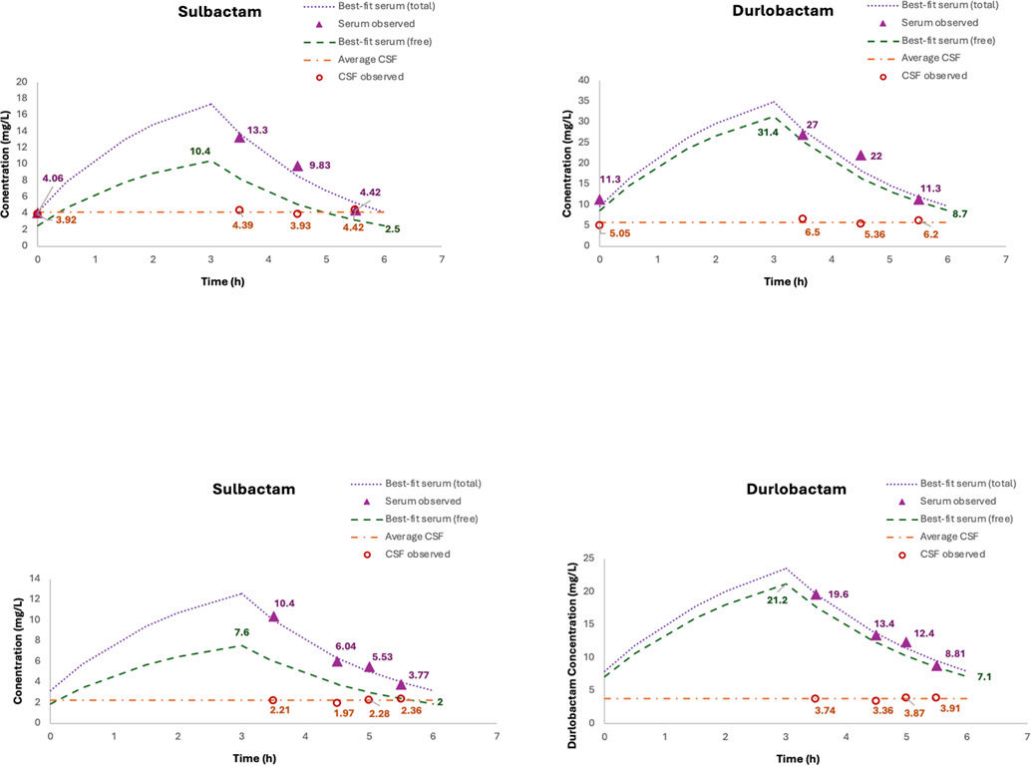
Observed and fitted serum and CSF concentrations at steady state. Both patients received sulbactam-durlobactam intravenously at a dose of 2 g every 6 h. Each dose is given as a prolonged infusion over 3 h. Samples were obtained immediately prior to the start of infusion, and at 0.5, 1.5, and 2.5 h after infusion completion for patient 1; and at 0.5, 1.5, 2, and 2.5 h after infusion completion for patient 2. *f*C_max_ and *f*C_min_ were derived from the best-fit parameter estimates.

The serum concentration-time profiles were well characterized (*r*² > 0.92, Fig. 1). In contrast, CSF drug concentrations remained largely constant throughout the dosing interval in both patients. Therefore, we did not model the CSF data as a fluctuating profile but instead reported the median concentration values. The best-fit pharmacokinetic parameters for sulbactam and durlobactam are presented in Table 1.

**TABLE 1:** Best-fit pharmacokinetic parameters

		Sulbactam		Durlobactam	
		Serum	CSF	Serum	CSF
Patient 1	Cl (L/h)	30.9	^*a*^-	15	-
	V_d_ (L)	65.3	-	35	-
	AUC total (mg.h/L)	64.7	-	133.4	-
	*f*AUC (mg.h/L)	38.8	25	120	34.07
	*f*Cmax (mg/L)	10.4	-	31.4	-
	*f*Cmin (mg/L)	2.5	-	8.7	-
	Half-life (h)	1.5	-	1.6	-
	CSF/serum penetration ratio		0.64		0.3
	R-squared	0.956		0.927	
Patient 2	Cl (L/h)	42.3	-	21.1	-
	V_d_ (L)	92.3	-	58.38	-
	AUC total (mg.h/L)	47.3	-	94.4	-
	*f*AUC (mg.h/L)	28.4	13.47	85	22.8
	*f*Cmax (mg/L)	7.6	-	21.2	-
	*f*Cmin (mg/L)	2	-	7.1	-
	Half-life (h)	1.5	-	2	-
	CSF/serum penetration ratio		0.47		0.27
	R-squared	0.982		0.974	

^*a*^ -, not applicable.

We present two cases of *A. baumannii* ventriculitis treated with sulbactam-durlobactam containing regimens, aiming to provide practical insights that may better guide clinicians in managing similar infections. A previously published case reported detectable but low CSF concentrations of the drugs (4). Unexpectedly low serum drug concentrations were likely due to augmented renal clearance, as observed in patient 2. This finding may support the need for a clinical dose adjustment to sulbactam-durlobactam 2 g every 4 h, in line with FDA guidance. However, limited pharmacokinetic insights were provided previously due to sparse pharmacokinetic sampling.

Our observations add valuable information to the limited literature on CNS penetration of sulbactam-durlobactam. As such, they offer a unique contribution to current knowledge on treatment options for severe CNS infections. Traditionally, CSF to serum area under the curve (AUC) ratios have been used to assess drug CNS penetration. However, if CSF concentrations remain stable over time, the AUC ratio becomes less informative. In both patients, we observed a relatively flat concentration-time profile in the CSF, indicating minimal fluctuation over time. Our data imply that a single CSF sample (either random or trough) at steady state may suffice to assess therapeutic adequacy. Without the knowledge of systemic exposure, comparing a single CSF concentration directly to the minimum inhibitory concentration (MIC) may offer a more convenient and clinically applicable approach for monitoring therapy.

There are several limitations to our study. We reported findings from only two cases; a larger and more diverse patient population would strengthen the results. Additionally, we did not correlate clinical outcomes with pharmacokinetic parameters, which could help inform optimal dosing strategies in the future. Finally, the mechanisms behind the observed steady-state CSF concentrations remain unclear. Mechanistic studies could further elucidate the CNS pharmacokinetics of sulbactam-durlobactam and similar antibiotics.

## References

[1] Karvouniaris M, Aidoni Z, Gkeka E, Primikyri SN, Pagioulas K, Argiriadou E. 2025. Treatment options for nosocomial ventriculitis/meningitis: a case report and review of the literature. Pathogens 14:3. doi:10.3390/pathogens14010003 PMC11768174 39860964

[2] Finch NA, Granillo A, Pouya N, Bhimraj A, Miller WR, Tam VH. 2025. Pharmacokinetics of cefiderocol in a patient with carbapenem-resistant Acinetobacter baumannii ventriculitis: a case report. Pharmacotherapy 45:66–69. doi:10.1002/phar.4632 39629915 PMC11757030

[3] Rodvold KA, Gotfried MH, Isaacs RD, O’Donnell JP, Stone E. 2018. Plasma and intrapulmonary concentrations of ETX2514 and sulbactam following intravenous administration of ETX2514SUL to healthy adult subjects. Antimicrob Agents Chemother 62:e01089-18. doi:10.1128/AAC.01089-18 30126953 PMC6201107

[4] Tamma PD, Immel S, Karaba SM, Soto CL, Conzemius R, Gisriel E, Tekle T, Stambaugh H, Johnson E, Tornheim JA, Simner PJ. 2024. Successful treatment of carbapenem-resistant Acinetobacter baumannii meningitis with sulbactam-durlobactam. Clin Infect Dis 79:819–825. doi:10.1093/cid/ciae210 38630890 PMC11478584

